# Acute remote home monitoring of acutely ill patients with COVID-19: how Dutch home monitoring initiatives were organized during the pandemic

**DOI:** 10.1186/s12913-024-11910-3

**Published:** 2024-12-02

**Authors:** Karin Smit, Rick T. van Uum, Stella Rijks, Alma C. van de Pol, Abeer Ahmad, Roderick P. Venekamp, Frans H. Rutten, Dorien L. M. Zwart, the Dutch General Practitioners Research Consortium

**Affiliations:** 1 Department of General Practice & Nursing Science, Julius Center for Health Sciences and Primary Care, University Medical Center Utrecht, Utrecht University, Utrecht, The Netherlands; 2 Department of General Practice, Amsterdam University Medical Center, Amsterdam, The Netherlands

**Keywords:** COVID-19, Telemonitoring, Remote monitoring, Home monitoring, Hospital at home, Acute home treatment

## Abstract

**Background:**

Acute remote home monitoring of acutely ill patients with COVID-19 holds potential for early detection of deterioration and thus subsequentearly intervention that may prevent or mitigate progression to severe illness and need for respiratory support. Our aim was to describe common features of acute remote home monitoring programs for acutely ill patients with COVID-19 in the Netherlands.

**Methods:**

We performed literature searches (both grey and academic) between 1st March 2020 and 1st March 2023 to identify Dutch acute remote home monitoring initiatives, excluding studies on early hospital discharge. From the available protocols, we extracted relevant information on patient eligibility, organization of acute remote home monitoring and home management.

**Results:**

We identified and approached ten acute remote home monitoring initiatives for information regarding their used protocols. Seven out of ten protocols were retrieved and assessed. All initiatives focused on adult patients with COVID-19 who where at risk of developing severe COVID-19, and all initiatives provided close follow-up through remote home monitoring using medically certified pulse oximeters. Daily measurements included peripheral oxygen saturation (all initiatives, *n* = 7), body temperature (*n* = 6), heart frequency per minute (*n* = 4) and breathing rate per minute (*n* = 4). For follow-up and review of measured values, in most initiatives (*n* = 6) the physician (general practitioner or hospital physician) in charge was supported by a dedicated monitoring center. In 5 out of 7 initiatives, the general practitioner (GP) was responsible for supervising the patients and monitoring staff.

**Conclusion:**

The acute remote home monitoring initiatives that emerged in the Netherlands during the first wave of the COVID-19 pandemic were similarly organized. Common building blocks for home monitoring include daily check of peripheral oxygen saturation, monitoring through a dedicated remote monitoring center alongside healthcare personnel and a supervising physician.

## Introduction

The COVID-19 pandemic and its pronounced impact on patient and healthcare resources spurred the development and implementation of remote monitoring initiatives [1]. The need to monitor patients with COVID-19 was particularly high during the first wave, as it was observed that patients could rapidly and unexpectedly deteriorate within the first seven to ten days [2]. This often led to the development of a critically low oxygen saturation level within a short timeframe, sometimes without -in first instance- accompanying shortness of breath and/or increased breathing rate, the so called silent or ‘happy’ hypoxaemia [3]. Remote home monitoring of oxygen saturation levels could capture early desaturation, thus allowing healthcare professionals to intervene before critical deterioration would occure, especially in patients with initially silent hypoxaemia. By safely managing patients with COVID-19 at home, remote monitoring made it possible to alleviate the pressure on both primary and secondary healthcare resources worldwide [4, 5].

Prior to the COVID-19 pandemic, remote home monitoring was mainly used to manage patients with stable chronic diseases known for exacerbations, e.g. chronic obstructive pulmonary disease and heart failure [6–8]. Thus, previous studies provided little scientific evidence on the feasibility and safety of remote monitoring in patients experiencing acute illness [9–11]. However, pulse oximetry was considered safe, and other general principles of remote monitoring for chronic diseases were deemed applicable to acute remote home monitoring during the pandemic.. This strategy was seen as a plausible alternative to straightforward hospitalizing these patients, especially driven by the shortage of hospital beds.

Remote home monitoring programs for acute care have now been developed in different healthcare systems worldwide [12–14]. The evaluation of such programs depends on how a healthcare system is organized. The Dutch health care system has a strong primary care practice, in which the general practitioner is the gatekeeper to hospital care. Thus, the vast majority of patients with COVID-19 were managed by general practitioners in the Netherlands. Eventually, just around 5% of patients with COVID-19 required hospital admission [15, 16].

We defined acute remote home monitoring as the remote monitoring of patients with COVID-19 if (i) this started within days after onset of symptoms, (ii) in those who were not yet hypoxaemic and (iii) therefore were not in need of oxygen therapy.

In this narrative review, we aimed to provide detailed information on acute home monitoring programs in the Netherlands, including outcomes wherever possible. We collected data from both published and unpublished programs, to prevent publication bias and contribute to the growing body of knowledge on remote care for acutely ill patients.

## Methods

### Search strategy and inclusion criteria

For identification of acute remote home monitoring initiatives in the Netherlands during the COVID-19 pandemic, we conducted both grey and academic literature searches between 1st March 2020 and 1st March 2023. For the grey literature search, we used search engines and websites including Google, ‘Medisch Contact’ (Dutch journal for medical professionals), Overton (policy documents), ‘Nederlands Tijdschrift voor Geneeskunde’ (Dutch journal for medical professionals), and the Nationwide Coordination Center for Patients with COVID-19 Spreading. The search terms included: home monitoring, tele monitoring, COVID-19, Netherlands. For the academic literature search, we used the following electronic databases: Pubmed, European Respiratory Journal, ClinicalTrials.gov, Cochrane Library, Embase, Scopus and UpToDate. Search terms included: home monitoring, remote monitoring, tele monitoring, COVID-19 and the Netherlands. In addition to the literature search, we contacted members of the Dutch General Practice Research Consortium via mail and/or telephone to further identify any initiatives that were not identified through our literature searches [13].

We included initiatives in which patients with COVID-19 were remotely monitored at home within days of onset of symptoms. We excluded initiatives that focused on monitoring patients after hospitalization for COVID-19 (i.e., early-discharge programs were not included).

### Search results

A total of ten remote home monitoring initiatives for acutely ill patients in different Dutch regions were identified. The research team reached out to the initiators of these home monitoring initiatives and received protocols from seven initiatives. Two of these initiatives had published their methods and results in a peer-reviewed journal [9, 17].

### Data collection and analyses

From the available protocols, relevant information was extracted regarding patient eligibility, organization of the home monitoring process, management of the patients and the used devices. The involved healthcare professionals were contacted to collect any missing information. Data were extracted independently by two research team members and any discrepancies were resolved by discussion.

From the two initatives that published their methods and results in a peer-reviewed journal, we extracted the following data: number of included patients, number of emergency department visits, hospital admission and deaths.

### Protocol availability

The protocols of the following organisations or networks were included:

1. *Assen* (cooperation of Wilhelmina Hospital Assen, Treant Care group Emmen/Hoogeveen/Meppel, and GP care Drenthe)
2. *Den Bosch* (Jeroen Bosch Hospital)
3. *Dordrecht* (Albert Schweitzer Hospital)
4. *Leiden* (Leiden UMC, Alrijne Hospital Leiderdorp and Reinier de Graaf Hospital Delft)
5. *Utrecht-1* (GP-driven initiative; CovidSat@Home, UMC Utrecht)
6. *Utrecht-2* (GP-driven initiative; CovidTherapy@Home, UMC Utrecht)
7. *Zwolle* (Isala Hospital)

The other three identified Dutch initiatives, one in the Northern and two from the Southern parts of the Netherlands were not available for review.

## Results

### Eligibility of patients

All seven available acute remote home monitoring protocols included eligibility criteria, which we summarized in Table 1. Home monitoring was generally limited to (i) patients with a positive polymerase chain reaction (PCR) test for COVID-19, except for one initiative; *Utrecht-1* which also included patients *suspected* of COVID-19*)*, (ii) patients without hypoxaemia (peripheral oxygen saturation levels ≥ 94%, breathing rate < 24/min, and no need for supplemental oxygen), and (iii) the presence of a family member or other care giver at home. Once again *Utrecht-1* was an exception, and a caregiver was not obligatory*.*

**Table 1:** Eligibility criteria Of the seven acute remote home monitoring initiatives of COVID-19 management

Eligibility criteria	Acute remote home monitoring initiatives								
	*Assen*	*Den Bosch*	*Dordrecht*	*Leiden*	*Utrecht-1*			*Utrecht-2*	*Zwolle*
COVID-19 diagnosis	PCR				Clinical^a^			PCR	
Age in years	≥ 18	≥ 18	≥ 18	≥ 18 AND < 80	≥ 40			≥ 18	> 70 years OR > 18 with comorbidity
Risk factors for deterioration	Yes	Yes	Yes	Yes	Yes			Yes	Yes
Additional requirements on comorbidities		BMI ≤ 30 kg/m^2^			Cardiovascular disease or CV risk factors				
Caregiver required	Yes	Yes	Yes	Yes	No			Yes	Yes
Proficiency in the Dutch language required	Yes								
Digital proficiency required	Yes					No	Yes		

*Abbreviations*: *BMI* body mass index, *CVD* cardiovascular disease, *CV* cardiovascular

^a^Moderate-to-severe COVID-19 symptoms defined as at least three days a body temperature ≥ 37.5 °C and either (i) new onset of symptoms of respiratory tract infection, and/or (ii) a feeling of shortness of breath, and/or (iii) a feeling of sudden exhaustion

All centers focussed the remote home monitoring on patients with increased risk of deterioration and thus considered in need of close follow-up because they might need hospitalization somewhat later in their disease trajectory. One center (*Assen*) focussed on patients at risk and especially on those who were frail.

In addition, *Utrecht-1* excluded patients with severe anaemia because of the risk of false low SpO_2_ readings when using pulse oximetry, and *Utrecht-2* excluded patients with severe dementia or psychiatric disorders (rendering patient incapable of adhering to study procedures). *Den Bosch/Dordrecht* excluded patients with concurrent pulmonary embolism (PE).

### Organization of acute home management

In two initiatives (*Dordrecht* and *Leiden*) *patients were* required to have visited the emergency department (ED) prior to inclusion in the acute home monitoring program and thus, in these centers, the hospital physician (mostly a pulmonologist) initiated the remote home monitoring. In *Assen, Utrecht-1* and *-2*, and *Zwolle,* the GP initiated the remote home monitoring while in *Den Bosch,* the GP could also initiate remote home monitoring without prior ED evaluation.

#### Medical therapy

Medical therapy for COVID-19 was left at the discretion of the patient’s own treating physician in all but one initiative; *Assen.* In that initiative, budesonide inhalation therapy was recommended based on the findings of the PRINCIPLE trial [18]. The GP was the patients’ primary responsible physician during the home management program in five initiatives (*Assen, Den Bosch, Utrecht-1, Utrecht-2 and Zwolle)*, while it was a hospital physician in *Dordrecht* (pulmonologist) and *Leiden* (internal medicine, pulmonologist).

### Organization of remote monitoring

#### Dedicated remote monitoring center

Most initiatives (*n* = 6) utilized a dedicated monitoring center staffed with trained personnel, including nurses, medical trainees, junior doctors, hospital physicians, or general practitioners. In *Utrecht-1,* the monitoring was performed by the patient’s own GP and the initiative did not use a dedicated monitoring center.

Patients were asked to register measurements of vital parameters in an app three times daily (*Assen*: four times daily). For *Utrecht-1,* only a paper diary was offered to register measurements. *Utrecht-2* offered a paper diary as an alternative to the digital monitoring app depending on patient’s preference.

Personnel from the monitoring centers contacted patients by phone if measurements were below or above pre-defined threshold values (*all initiatives except Utrecht-1*) and some also contacted patients on fixed times every day irrespective of the measurement values (*Den Bosch, Leiden, Utrecht-2*). For *Utrecht-1*, patients were instructed to call the GP themselves if they thought it was necessary (Table 2).

**Table 2:** Measurements and Action Thresholds for Patient Intervention and Contact

Measurement	Remote home monitoring initiatives						
	*Assen*	*Den Bosch*	*Dordrecht*	*Leiden*	*Utrecht-1*	*Utrecht-2*	*Zwolle*
Peripheral oxygen saturation (%)	≤ 90 (red alert)^b^ ≤ 92 (orange alert) ^b^ Drop ≥ -2	≤ 89 (red alert) ^b^ ≤ 93 (orange alert) ^b^	≤ 91 (red alert)^b^	individual threshold^a^	< 94	< 94	< 92
Breathing rate per minute	≥ 24	≥ 20 or + 2^c^	≥ 20	individual threshold^a^			
Ear temperature, (°C)	≥ 38.5	> 37.9	≥ 38.5	individual threshold^a^		≤ 35.9 or ≥ 38.0	
Heart rate in beats per minute	≥ 100			individual threshold^a^	No threshold defined	≤ 49 or ≥ 101	
Cough score (0–10 points)		> 4 or + 2				≥ 8 and/or + 2	
Shortness of breath score (0–10 points)		> 5 or + 2				≥ 5 and/or + 2	
Shortness of breath (yes/no)		if YES				if YES	
General wellbeing (better (positive) or worse (negative) in comparison to day before)	If negative	If negative				If negative	
If value was not reported	Oxygen saturation, heart rate, breathing rate		Any				
Blood pressure in mmHg				individual threshold^a^			

^a^Individual thresholds were determined by the treating physician based on the patient’s medical history, and these may vary from general thresholds

^b^Red alert means contacting directly the patient; orange alert means that the patient is asked for re-measurement after a couple of minutes and if values are below the threshold than being upscaled to red alert

^c^per 30 s

#### Measurements

As shown in Table 2, these measurements consisted of:

- Peripheral oxygen saturation with pulse-oximetry: *all initiatives*
- Breathing rate per minute: *Assen, Den Bosch, Dordrecht, Leiden*
- Body temperature, ear in Celsius: all except *Utrecht-1*
- Heart rate in beats per minute: *Assen, Leiden, Utrecht-1* and *Utrecht-2*
- Cough score (self-reported scale or increase of cough): *Den Bosch, Utrecht-2*
- Shortness of breath (SOB) score (self-reported scale or increase of SOB): *Den Bosch, Utrecht-2*
- General wellbeing (better (positive) or worse (negative) as compared to the day before): *Assen, Den Bosch, Utrecht-2*
- Blood pressure in mmHg: *Leiden*

All initiatives established prespecified thresholds for measurement values. In *Den Bosch, Utrecht-1* and *Utrecht-2,* the threshold for peripheral oxygen saturation was set at 94%, in *Assen* at 93% and in both *Dordrecht* and *Zwolle* at 92%. In Leiden, the protocol set individual alarm thresholds for oxygen saturation levels, based on medical history. Three initiatives (*Assen, Den Bosch and Dordrecht*) used orange and red alerts to differentiate theseverity of desaturation, as measured with a peripheral pulse oximeter. In *Utrecht-1,* patients were instructed to contact their own GP directly in case of persistent low peripheral oxygen saturation levels (two consecutive measurements < 94%). For all other initiatives, this was monitored real-time or at set intervals by the monitoring center digitally (Table 2).

#### Monitoring during out-of-hours

Monitoring centers were generally operating during working hours, but *Assen and Dordrecht* continued until 10 and 9 PM, respectively. In case of emergency after these hours, patients could contact the out of hours primary care (OOH PC) services. *Zwolle* offered an emergency contact line in case the GP or the OOH -GP services could not be reached by the patient or his/her informal caregiver.

#### Termination of remote monitoring

Remote monitoring was terminated if a patient’s oxygen saturation level remained stable for 24 h (*Zwolle*), 48 h (*Assen*) or 7 days (*Dordrecht*), or at discretion of the GP (*Den Bosch, Utrecht-2*). For *Utrecht-1* (trial setting), patients were standardly monitored for 14 consecutive days.

### Devices used

The pulse oximeters used by the initatives were provided to the patient through various means; via the emergency department (*Den Bosch, Dordrecht*), the general practitioner (*Assen*), the monitoring center (*Zwolle*) or the researcher (*Utrecht-1 and -2*). In one center (*Leiden*) patients received at the emergency department a box containing a pulse oximeter, thermometer and blood pressure measuring device. All centers provided medically certified pulse oximeters: HUM Aerocheck (*Assen*), Medisana (*Den Bosch),* HbO-Smart Drive DeVilbiss (*Dordrecht*), Masimo MightySatRx (*Leiden),* iHealth air (*Utrecht-2, Zwolle*), and Nonin type 3230 (*Utrecht-1, Utrecht-2).* Patients were in general required to use their own (ear) thermometer, except for *Leiden* (Withings Thermo), and *Utrecht-2* (Braun Thermoscan 7).

#### Patient outcomes from two of the Dutch initiatives

Only two of the Dutch initiatives (*Utrecht-1*, a pilot RCT; *Leiden,* a case–control study) published patient outcomes of 41 and 55 patients with COVID-19, respectively [9, 17]. The patients in the *Utrecht-*1 pilot RCT compared usual care (no remote monitoring) to usual care plus remote home monitoring with medically certified pulse oximetry. Patients randomised to the intervention arm using a pulse oximeter reported a significantly higher ‘feeling of safety’ than those allocated to usual care. A total of 5 patients from the intervention group versus 1 patient from the usual care group required hospital admission [9].

In *Leiden*, a retrospective case–control study was performed to evaluate the home monitoring program; 55 patients who received home monitoring were compared to 110 ‘propensity score matched controls’. Allocation was based on the ED physician’s clinical judgement and the groups significantly differed in the number of patients with confirmed COVID/19; 16 (29%) versus 9 (8%), *p* < 0.001, respectively. Five patients from the home monitoring group (9%) required hospital admission during follow-up versus 30 patients from the control group (27%), 25 out of those 30 admissions (87%) were so called short-stay admission (discharge < 24 h) without invasive in-hospital treatment [17].

None of the 96 patients died in either study while receiving acute home monitoring, but both studies were too small to draw meaningful conclusions on risk of hospital admission or mortality.

## Discussion

### Summary of main findings

The acute remote home monitoring initiatives that emerged in the Netherlands during the first wave of the COVID-19 pandemic were similarly organized. Common building blocks of these programs for acute home monitoring included daily checks of peripheral oxygen saturation, monitoring through a dedicated remote monitoring center, and close collaboration between healthcare personnel and supervising physician.

### Comparison to literature

#### Patients at risk

The identified Dutch initiatives focussed on remote home monitoring of patients with COVID-19 at risk of deterioration. In the Netherlands, only 5% of patients infected with SARS-CoV-2 required hospital admission for further treatment, highlighting that remote monitoring may be limited to those at high risk hospitalisation [16].

In the United Kingdom, a similar home monitoring program, COVID Oximetry@Home, was developed and implemented at large scale during the pandemic. The program was directed at monitoring patients with a risk of deterioration, such as patients ≥ 65 years of age or younger patients with a higher risk of complications from COVID-19. Patients were usually referred to the program by a GP. Patients received a pulse oximeter to measure peripheral oxygen saturation three times a day and healthcare professionals, including GPs, contacted patients to assess symptoms and vital signs. Usually, patients were monitored for 14 days [19].

In contrast to focusing on patients at high risk from COVID-19, a program in the United States, a large-scale COVID-19 surveillance system called COVID Watch, enrolled all patients who tested positive for COVID-19, regardless of symptoms. The patients received twice daily an automated text message to assess how they were feeling and whetherthey were experiencing breathing difficulties This was automated and included a protocol for escalation. In a large-scale randomized controlled trial performed alongside COVID Watch, pulse oximetry was added to the monitoring [11, 20]. Among patients with COVID-19, the addition of home pulse oximetry to remote monitoring did not result in a greater number of days alive and out of the hospital than subjective assessments of dyspnea alone.

#### Detection of hypoxaemia

The peripheral oxygen saturation thresholds for alarm varied across the Dutch remote home monitoring initiatives, ranging from < 94% to ≤ 91%. This variation might be attributed to the lack of strict criteria and guidelines especially during the first COVID-19-wave. The Dutch primary care practice guideline ‘Acute Coughing’ recommended that GPs should at least contact a pulmonologist (or internal medicine specialist) if oxygen levels fell to 92–94% or lower [21, 22]. In general, patients with oxygen levels < 90% require supplementary oxygen to prevent serious hypoxaemia-induced damage [23–25].

#### Building blocks and common elements of remote monitoring

The above mentioned programs from the UK and US contained similar elements as our Dutch initiatives, as well as other remote home monitoring programs identified in a systematic review of Vindrola-Padros et al. [15] This review of Vindrola-Padros et al. identified 13 publications from seven countries on initiatives directed to monitor patients at home for COVID-19. In the review of Vindrola-Padros et al., the results shows that most remote monitoring programs offered daily symptoms (notably level of shortness of breath) via digital forms or phone contact. In 54% (7/13) of the studies, patients also received a pulse oximeter for regularly daily oxygen saturation level checks, and 46% (6/13) of the studies also used daily temperature monitoring. These findings align with the Dutch program’s focus on regular daily symptom checks. In the review of Vindrola-Padros et al. most programs had a follow-up strategy that was based on risk stratification considering the mentioned monitoring items. Patients who tested positive for COVID-19 but did not have any symptoms were assessed only once. This is different from our narrative review of Dutch initiatives in which only patients at high risk of developing severe illness were included.

In the included programs of the review of Vindrola-Padros et al., remote monitoring and assessment of the data was performed by trained healthcare staff, often nurses or physicians from a hospital (7/13), and was scheduled differently in studies, ranging from only once daily to three times daily. In contrast to our review, most programs (77%, *n* = 10/13) identified in the international review were hospital led.

In all, common elements across all programs on acute remote home monitoring initiatives for COVID-19 patients included regular re-assessment of the (adult) patient by a team of healthcare professionals based on a combination of subjective symptoms and vital signs, with peripheral oxygen saturation as the most important measurement to detect hypoxemia.

#### Strengths and limitations

In this narrative review we provided additional and more detailed information on acute home monitoring programs in the Netherlands, including outcomes wherever possible. A strength is that we could assess seven independent initiatives offered in a single healthcare system by retrieving the protocols and asking the initiators for additional information, thus having richer data than previous reviews of remote home monitoring initiatives. By including programs not published, we tried to prevent publication bias. Despite our best efforts, it is possible we may not have identified all initiatives. However, given the similarities among the initiatives included in the review, we think that the inclusions of additional initiatives would not have yielded different conclusions.

A limitation is that we were not able to evaluate patient-relevant outcome data for most of the initiatives identified in this study, thus, leaving the impact of these monitoring initiatives on numbers of healthcare resource use and other healthrelated outcomes largely unknown. To foster change, it is key to assess which home remote monitoring ‘common building blocks’ are most useful. To this end, we encourage data sharing of patient outcomes through nationwide collaboration.

#### Implications for clinical practice

This study illustrates the organization of Dutch acute remote home monitoring initiatives as they were developed and implemented during the COVID-19 pandemic. It provides an overview of consistent elements for home monitoring, which can serve as a framework for further implementing remote home monitoring, including other conditions than COVID-19, and manage acute phases of more chronic diseases. For instance, while patients with chronic obstructive pulmonary disease are already being monitored in their stable phase to detect exacerbation early, a remote home monitoring framework could also be adapted to manage acute exacerbations at home.

Albeit all initiatives used monitoring of vital signs to detected deterioration, the type and brand of the devices used to measure these vital signs differed. Especially pulse oximeters play a crucial role in remote monitoring by assessing peripheral oxygen saturation which is a key indicator of deterioration. The initiatives in this review used pulse oximeters of different types and brands, ranging from non-medically validated devices to FDA/ISO approved ones. Importantly, there was a shortage of validated pulse oximeters during the first wave of COVID-19 in the Netherlands. The variability in pulse oximeters highlights the importance of selecting those devices that are validated and reliable to detect deterioration. Future research should prioritize the determination of the most cost-effective and reliable methods to detect deterioration and include an evaluation of pulse oximeters to establish minimum standards necessary for safe and effective acute remote monitoring. Moreover, future research should also evaluate the value of patients self-reported items such as shortness of breath or worsening condition. Finally, our results may be transferable to other countries with similar healthcare system where collaboration between GPs and hospital physicians could together in cooperative care contribute to safe and effective remote home monitoring.

## Conclusions

The acute remote home monitoring initiatives that emerged in the Netherlands during the first wave of the COVID-19 pandemic were similarly organized. Generic building blocks for home monitoring included daily checks of peripheral oxygen saturation, centralized monitoring via a dedicated remote monitoring center and close collaboration between healthcare personnel and supervising physician. This structured approach seems to ensure the necessary continuity of care in the critical first days of patients with COVID-19. The initiatives highlight the importance of collaborative care and how to use and integrate remote home monitoring into healthcare models and structures.

## Data Availability

The dataset generated during and analysed during the current study are available upon request.

## Abbreviations

BMI: Body Mass Index
COPD: Chronic Obstructive Pulmonary Disease
COVID-19: Coronavirus Disease 2019
ED: Emergency Department
GP: General Practitioner
IQR: Interquartile Range
L/min: Liters per minute
OOH: Out-of-Hours
PCR: Polymerase Chain Reaction
RCT: Randomized Controlled Trial
UMC: University Medical Center

## References

[1] Monaghesh E, Hajizadeh A. The role of telehealth during COVID-19 outbreak: a systematic review based on current evidence. BMC Public Health. 2020;20(1):1193. 32738884 10.1186/s12889-020-09301-4 PMC7395209

[2] Montazersaheb S, Hosseiniyan Khatibi SM, Hejazi MS, Tarhriz V, Farjami A, Ghasemian Sorbeni F, et al. COVID-19 infection: an overview on cytokine storm and related interventions. Virol J. 2022;19(1):92. 35619180 10.1186/s12985-022-01814-1 PMC9134144

[3] Jouffroy R, Jost D, Prunet B. Prehospital pulse oximetry: a red flag for early detection of silent hypoxemia in COVID-19 patients. Crit Care. 2020;24(1):313. 32513249 10.1186/s13054-020-03036-9 PMC7278215

[4] Banzi R, Sala L, Colmi A, Gerardi C, Nattino G, Occhipinti F, et al. Feasibility and efficacy of home monitoring for patients with suspected or confirmed COVID-19. Recenti Prog Med. 2020;111(10):584–92. 33078008 10.1701/3453.34418

[5] Al-Tawfiq JA, Kheir H, Al-Dakheel T, Al-Qahtani S, AlKhadra H, Sarhan A, et al. COVID-19 home monitoring program: Healthcare innovation in developing, maintaining, and impacting the outcome of SARS-CoV-2 infected patients. Travel Med Infect Dis. 2021;43:102089. 34087448 10.1016/j.tmaid.2021.102089 PMC8169224

[6] Bashi N, Karunanithi M, Fatehi F, Ding H, Walters D. Remote Monitoring of Patients With Heart Failure: An Overview of Systematic Reviews. J Med Internet Res. 2017;19(1):e18. 28108430 10.2196/jmir.6571 PMC5291866

[7] Barbosa MT, Sousa CS, Morais-Almeida M, Simões MJ, Mendes P. Telemedicine in COPD: An Overview by Topics. COPD. 2020;17(5):601–17. 32892650 10.1080/15412555.2020.1815182

[8] Hanlon P, Daines L, Campbell C, McKinstry B, Weller D, Pinnock H. Telehealth Interventions to Support Self-Management of Long-Term Conditions: A Systematic Metareview of Diabetes, Heart Failure, Asthma, Chronic Obstructive Pulmonary Disease, and Cancer. J Med Internet Res. 2017;19(5):e172. 28526671 10.2196/jmir.6688 PMC5451641

[9] Smit K, Venekamp RP, Krol LA, Geersing G-J, Schoonhoven L, Kaasjager KA, et al. Home monitoring by pulse oximetry of primary care patients with COVID-19: a pilot randomised controlled trial. Br J Gen Pract. 2023:BJGP.2022.0224. 10.3399/BJGP.2022.0224 PMC9926282 37105736

[10] Alboksmaty A, Beaney T, Elkin S, Clarke JM, Darzi A, Aylin P, Neves AL. Effectiveness and safety of pulse oximetry in remote patient monitoring of patients with COVID-19: a systematic review. Lancet Digit Health. 2022;4(4):e279–89. 35337644 10.1016/S2589-7500(21)00276-4 PMC8940208

[11] Lee KC, Morgan AU, Chaiyachati KH, Asch DA, Xiong RA, Do D, et al. Pulse Oximetry for Monitoring Patients with Covid-19 at Home - A Pragmatic Randomized Trial. N Engl J Med. 2022;386(19):1857–9. 35385625 10.1056/NEJMc2201541 PMC9006781

[12] Morgan AU, Balachandran M, Do D, et al. Remote monitoring of patients with Covid-19: design, implementation, and outcomes of the first 3,000 patients in COVID Watch. NEJM Catalyst. 2020. https://catalyst.nejm.org/doi/full/10.1056/CAT.20.0342.

[13] Nachega JB, Leisegang R, Kallay O, Mills EJ, Zumla A, Lester RT. Mobile Health Technology for Enhancing the COVID-19 Response in Africa: A Potential Game Changer? Am J Trop Med Hyg. 2020;103(1):3–5. 32476643 10.4269/ajtmh.20-0506 PMC7356462

[14] Greenhalgh T, Knight M, Inada-Kim M, Fulop NJ, Leach J, Vindrola-Padros C. Remote management of covid-19 using home pulse oximetry and virtual ward support. BMJ. 2021;372:n677. 33766809 10.1136/bmj.n677

[15] Vindrola-Padros C, Singh KE, Sidhu MS, Georghiou T, Sherlaw-Johnson C, Tomini SM, et al. Remote home monitoring (virtual wards) for confirmed or suspected COVID-19 patients: a rapid systematic review. eClinicalMedicine. 2021;37:100965. 34179736 10.1016/j.eclinm.2021.100965 PMC8219406

[16] van Royen FS, Joosten LPT, van Smeden M, Slottje P, Rutten FH, Geersing GJ, van Doorn S. Cardiovascular vulnerability predicts hospitalisation in primary care clinically suspected and confirmed COVID-19 patients: A model development and validation study. PLoS ONE. 2022;17(4):e0266750. 35404964 10.1371/journal.pone.0266750 PMC9000124

[17] Dirikgil E, Roos R, Groeneveld GH, Heringhaus C, Silven AV, Petrus AHJ, Villalobos-Quesada M, Tsonaka R, van der Boog PJM, Rabelink TJ, Bos WJW, Chavannes NH, Atsma DE, Teng YKO. Home monitoring reduced short stay admissions in suspected COVID-19 patients: COVID-box project. Eur Respir J. 2021;58(2):2100636. 10.1183/13993003.00636-2021. 10.1183/13993003.00636-2021 PMC8015644 33795321

[18] Yu LM, Bafadhel M, Dorward J, Hayward G, Saville BR, Gbinigie O, et al. Inhaled budesonide for COVID-19 in people at high risk of complications in the community in the UK (PRINCIPLE): a randomised, controlled, open-label, adaptive platform trial. Lancet. 2021;398(10303):843–55. 34388395 10.1016/S0140-6736(21)01744-X PMC8354567

[19] England NHSN. COVID-19 guidance note: COVID Oximetry@home. 2022.

[20] Delgado MK, Morgan AU, Asch DA, Xiong R, Kilaru AS, Lee KC, et al. Comparative Effectiveness of an Automated Text Messaging Service for Monitoring COVID-19 at Home. Ann Intern Med. 2022;175(2):179–90. 34781715 10.7326/M21-2019 PMC8722738

[21] De Bont EGPM GJ, Kurver MJ, Van der Moorden FM, Van de Pol AC, Prins JM, Schot MJC, Verheij ThJM, Weersma RLS. Dutch GP Guidelines: Acuut Hoesten 2011 updated 2024. 2024: Available from: https://richtlijnen.nhg.org/standaarden/acuut-hoesten.

[22] El Bouazzaoui L BM, Cals J, Geerlings S, Geersing G, Greving J, Kok-Pigge A, Kuijpers T, Loogman M, Nijs M, Otto-Paling F, Platteel F, Verheij T. Dutch GP Guidelines: COVID-19 2021 updated 2023. 2023: Available from: https://richtlijnen.nhg.org/standaarden/covid-19.

[23] Hardinge M, Annandale J, Bourne S, Cooper B, Evans A, Freeman D, et al. British Thoracic Society guidelines for home oxygen use in adults. Thorax. 2015;70(Suppl 1):i1-43. 25870317 10.1136/thoraxjnl-2015-206865

[24] Ekström M, Ringbaek T. Which patients with moderate hypoxemia benefit from long-term oxygen therapy? Ways forward. Int J Chron Obstruct Pulmon Dis. 2018;13:231–5. 29386891 10.2147/COPD.S148673 PMC5765977

[25] Luks AM, Swenson ER. Pulse Oximetry for Monitoring Patients with COVID-19 at Home. Potential Pitfalls and Practical Guidance. Ann Am Thorac Soc. 2020;17(9):1040–6. 32521167 10.1513/AnnalsATS.202005-418FR PMC7462317

